# Evaluation of the Elecsys Chagas Assay for Detection of Trypanosoma cruzi-Specific Antibodies in a Multicenter Study in Europe and Latin America

**DOI:** 10.1128/JCM.01446-17

**Published:** 2018-04-25

**Authors:** Maria Delmans Flores-Chavez, Vittorio Sambri, Volkmar Schottstedt, Fernando Aparicio Higuera-Escalante, Dieter Roessler, Monica Chaves, Tina Laengin, Alfredo Martinez, Bernhard Fleischer

**Affiliations:** a Unit of Leishmaniasis and Chagas Disease, Department of Parasitology, National Microbiology Centre (CNM), Instituto de Salud Carlos III, Madrid, Spain; b Unit of Microbiology, Greater Romagna Hub Laboratory, AUSL della Romagna, Pievesestina, Cesena, Italy; c DIMES, University of Bologna, Bologna, Italy; d German Red Cross Blood Transfusion Service West, Central Laboratory, Hagen, Germany; e Blood Bank Higuera-Escalante, Bucaramanga, Colombia; f Roche Diagnostics GmbH, Penzberg, Germany; g Productos Roche SA, Bogotá, Colombia; h Centro de Educación Médica e Investigaciones Clínicas Dr. Norberto Quirno, Buenos Aires, Argentina; i National Reference Centre for Tropical Infectious Agents, Bernhard Nocht Institute for Tropical Medicine, Hamburg, Germany; University of Tennessee at Knoxville

**Keywords:** Chagas disease, electrochemiluminescence immunoassay, serology, Trypanosoma cruzi

## Abstract

Serology is the preferred method to confirm a Chagas disease diagnosis and to screen blood donors. A battery of assays is often required due to the limited accuracy of single assays. The Elecsys Chagas assay is a newly developed, double-antigen sandwich assay for use on the Elecsys and cobas e immunoassay analyzers, intended to identify individuals infected with Trypanosoma cruzi, for diagnosis and screening. The performance of the Elecsys Chagas assay was evaluated in comparison with those of other widely used T. cruzi antibody assays, at multiple sites (Europe/Latin America). Relative sensitivity and specificity were assessed by using samples from blood donors, pregnant women, and hospitalized patients from regions where Chagas disease is endemic and from regions of nonendemicity. The Elecsys Chagas assay had an overall relative sensitivity of 100% (*n* = 674). Overall relative specificities were 99.90% (*n* = 14,681), 100% (*n* = 313), and 100% (*n* = 517) for samples from blood donors, pregnant women, and hospitalized patients, respectively. The analytical specificity was 99.83% (*n* = 594). The Elecsys Chagas assay detected T. cruzi antibodies in two World Health Organization (WHO) standard T. cruzi reference panels (panels 09/188 and 09/186) at a 1:512 dilution, corresponding to a cutoff sensitivity of approximately 1 mIU/ml. The Elecsys Chagas assay demonstrated robust performance under routine conditions at multiple sites in Europe and Latin America. In contrast to other available Chagas assays, the Elecsys assay uses a reduced number of recombinant T. cruzi antigens, resulting in a significantly smaller number of cross-reactions and improved analytical specificity while being highly sensitive.

## INTRODUCTION

Chagas disease (American trypanosomiasis) is caused by the flagellate protozoan Trypanosoma cruzi and affects 6 million to 7 million people worldwide, mainly in Latin America (1, 2). Vector-borne transmission via insects of the subfamily Triatominae occurs in the Americas; however, infection can also be transmitted congenitally and via blood transfusion, organ transplantation, and the ingestion of food/beverages contaminated with parasites (3–6). Although it mainly affects individuals living in regions of endemicity, the disease has now spread to other regions and continents (7–10).

The infection is characterized by an acute, often asymptomatic stage lasting 8 to 12 weeks, when active parasitemia is evident. During this stage, diagnosis can be performed by direct microscopy of blood for circulating parasites or via PCR. The infection subsequently enters a chronic phase (1, 11, 12), and the majority of individuals remain asymptomatic. However, up to 30% of individuals will develop Chagas cardiomyopathy over decades, and up to 10% will develop gastrointestinal, neurological, or oligosymptomatic alterations that require treatment (1, 11, 12); these pathologies typically develop over years to decades. Generally, in the chronic phase of infection, the management of specific symptoms/conditions is necessary (13–15). The availability of curative treatment is a controversial topic; the WHO recommends treatment of adults with antiparasitic drugs to prevent disease progression and congenital transmission in pregnant women (1).

Importantly, chronically infected individuals represent a substantial population capable of transmitting the infection, particularly through blood or organ donation or from mother to child (4, 6). During the chronic phase, individuals exhibit low-level or no parasitemia, and thus, direct microscopy is inappropriate. PCR detects the parasite in 40 to 65% of patients with chronic disease (16, 17); consequently, diagnosis relies upon the detection of T. cruzi antibodies by serological methods (13, 17, 18).

The most commonly used serological methods are enzyme-linked immunosorbent assay (ELISA) and immunofluorescence indirect test (IFI) (13, 17, 19); a few automated systems based on chemiluminescence have been introduced (38, 40). The diagnostic approach to Chagas disease is heterogeneous, with guidelines varying according to location (i.e., laboratory, region, or country) and purpose (i.e., screening of blood/organ donors versus diagnosis of patients with symptomatic disease) (22–26). Conventional tests based on total antigens show cross-reactivity between T. cruzi and Leishmania spp. or Trypanosoma rangeli; therefore, confirmation of the presence of T. cruzi antibodies commonly requires the use of at least two tests that are based on different methods/antigens. Furthermore, the resolution algorithms (i.e., sequence, type, and number of tests used) also vary by region (22, 25, 27). However, with the availability of assays with improved sensitivity and specificity, it has been suggested that a single assay may now be adequate for screening and diagnosis (28).

The primary aim of this study was to assess the relative sensitivity and specificity of the fully automated Elecsys Chagas assay in comparison with those of other state-of-the-art T. cruzi antibody assays. Secondary aims were the evaluation of analytical specificity and analytical sensitivity at the cutoff (CO) in dilution series against WHO standards.

## MATERIALS AND METHODS

### Study design.

The analytical performance of the Elecsys Chagas assay was evaluated at five independent laboratories in Europe and Latin America and at the Roche Diagnostics assay development facility. The study was performed between August 2015 and July 2016. All samples were anonymized or pseudonymized residual fresh or frozen serum/plasma samples from either daily routine or blood donor testing (Table 1).

**TABLE 1:** Sample cohorts and assays used for relative sensitivity and specificity evaluation

Site	Cohort type	No. of samples	Source	Condition	Matrix	Comparator assay tested
Relative specificity						
Hagen, Germany	Blood donors	4,391	Blood screening	Fresh	Serum	Abbott Prism Chagas
Pievesestina, Italy	Blood donors	5,244	Blood screening	Fresh	Serum	Ortho T. cruzi ELISA; DiaSorin Liaison XL Murex
	Hospitalized patients	500	Daily routine	Frozen	Serum	DiaSorin Liaison XL Murex
	Pregnant women	239	Daily routine	Frozen	Serum	DiaSorin Liaison XL Murex
Bucaramanga, Colombia	Blood donors	2,707	Blood screening	Fresh	Plasma	Abbott Architect Chagas
Buenos Aires, Argentina	Blood donors	1,056	Blood screening	Fresh	Serum	Abbott Architect Chagas
	Blood donors	1,283	Blood screening	Frozen	Serum	Abbott Architect Chagas
	Hospitalized patients	17	Daily routine	Fresh	Serum	Abbott Architect Chagas
	Pregnant women	74	Daily routine	Fresh	Serum	Abbott Architect Chagas
Relative sensitivity						
Madrid, Spain	Chagas positive	500	Collection of stored samples	Frozen	Serum/plasma	Ortho T. cruzi ELISA
Buenos Aires, Argentina	Chagas positive^*a*^	174	Serotheque	Frozen	Serum	Abbott Architect Chagas

a Samples collected at the Universidad Nacional del Litoral, Santa Fe, Argentina.

Prior to the start of the study, ethical approval (or waiver) was obtained from the relevant local authorities. The study was conducted in accordance with the principles of the Declaration of Helsinki and International Conference on Harmonisation guidelines for good clinical practice. Where necessary, donors/participants provided written informed consent.

### Elecsys Chagas and comparator assays.

The Elecsys Chagas assay is an automated electrochemiluminescence immunoassay for the qualitative determination of antibodies to T. cruzi for use on cobas e analyzers (clinical chemistry analyzer and immunochemistry analyzer) in equipped laboratory settings. The assay is based on a double-antigen sandwich principle, utilizing soluble forms of recombinant T. cruzi antigens derived from flagellar calcium binding protein, flagellar repetitive antigen, and cruzipain (the major cysteine proteinase of T. cruzi).

During the first incubation, 18 μl of a sample is added to a reaction mixture containing biotin-labeled and ruthenium-labeled T. cruzi antigens to form antibody-antigen immune complexes (with one antigen binding site of the patient's specific immunoglobulin G [IgG] binding the biotinylated antigen and the other paratope binding the ruthenium-labeled antigen). In a second incubation, the IgG–double-antigen sandwich complex is bound via biotin to streptavidin-coated beads and subsequently transferred to the measuring cell, where the microparticles are magnetically captured on the surface of the electrode. Chemiluminescent emission from the ruthenium label is induced by the application of voltage to the electrode and measured by using a photomultiplier. The results are automatically determined by software based on comparison of the electrochemiluminescence signal obtained from the reaction product with the cutoff value previously obtained during system calibration. The total assay time is 18 min.

The following comparison assays were performed according to the manufacturers' recommendations (including calibration and the respective control runs): whole-cell lysate Ortho T. cruzi ELISA (Ortho Clinical Diagnostics, Johnson & Johnson, High Wycombe, UK), Abbott Architect Chagas and Abbott Prism Chagas (Abbott Diagnostics, IL, USA), DiaSorin Liaison XL Murex Chagas (DiaSorin SpA, Saluggia, Italy), Wiener Lab Chagatest ELISA recombinante v.4.0 (Wiener Lab Group, Rosario, Argentina), NovaTech NovaLisa Chagas (NovaTech Immundiagnostica GmbH, Dietzenbach, Germany), and Biokit Bioelisa Chagas IgG (Biokit SA, Barcelona, Spain).

### Relative sensitivity.

The relative sensitivity of the Elecsys Chagas assay was evaluated at sites in Europe (*n* = 1) and Latin America (*n* = 1) by using precharacterized Chagas disease-positive samples obtained from Chagas disease-infected patients in both regions. Comparison assays were the whole-cell-lysate Ortho T. cruzi ELISA and the Abbott Architect Chagas assay. Samples tested with the Ortho T. cruzi ELISA were previously characterized by using in-house assays (ELISA-Centro Nacional de Microbiología [CNM] and IFI-CNM) (29), Wiener Lab Chagatest ELISA recombinante v.4.0, and PCR (30), while samples tested with the Abbott Architect Chagas assay were previously characterized with at least two of the following serology assays: Wiener Lab Chagatest ELISA recombinante v. 4.0, Wiener Lab Chagatest hemagglutination indirect test (HAI), and in-house IFI (antigens and controls were provided by the National Institute of Parasitology Dr. Mario Fatala Chaben, Buenos Aires, Argentina). Tests for samples with nonreactive results in the comparison assay were repeated in triplicate.

### Relative specificity.

The relative specificity of the Elecsys Chagas assay was evaluated at four sites in Europe (*n* = 2) and Latin America (*n* = 2), using samples from blood donors, pregnant women, and hospitalized patients obtained in both regions.

For the testing of samples from blood donors, comparison assays were the Ortho T. cruzi ELISA, the DiaSorin Liaison XL Murex Chagas assay, the Abbott Prism Chagas assay, and the Abbott Architect Chagas assay. For the testing of samples from pregnant women and hospitalized patients, comparison assays were the DiaSorin Liaison XL Murex Chagas and the Abbott Architect Chagas assays.

Initial determinations were carried out with single measurements. Samples with discrepant and concordant reactive results were repeated in duplicate for the respective assays and were considered to be repeatedly reactive (RR) if either of the retest results had a signal/cutoff ratio of ≥1.00.

**FIG 1:**
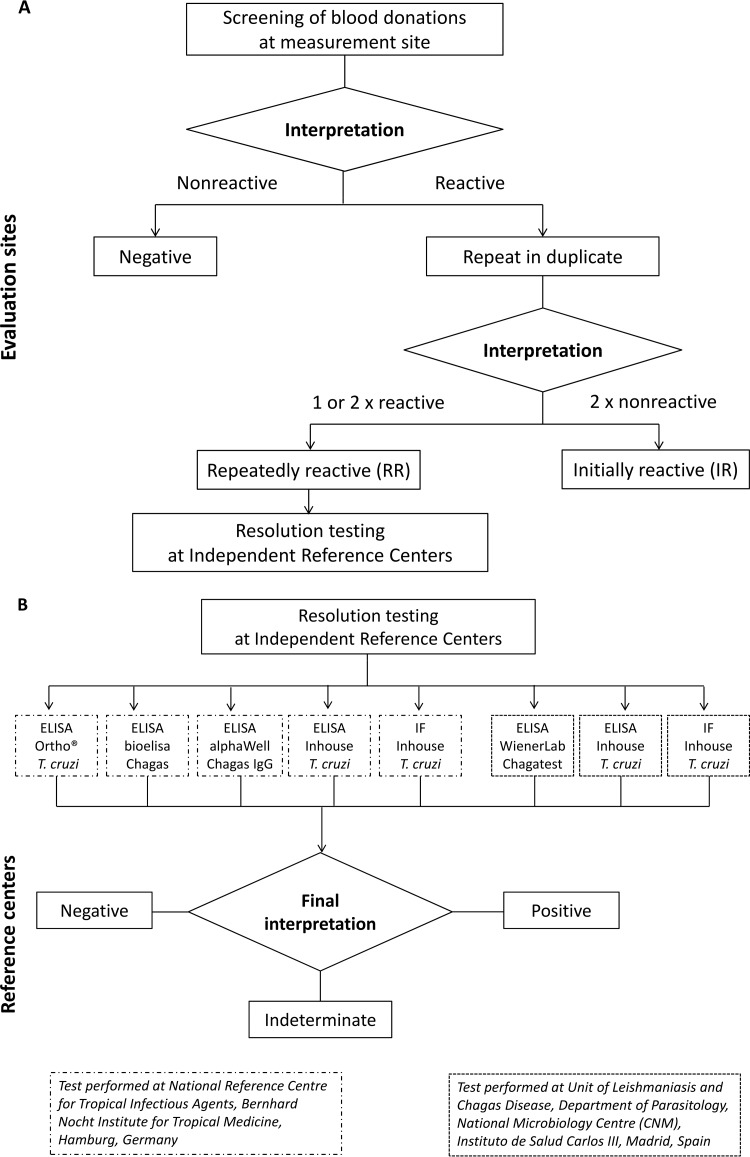
(A) Sample workflow for evaluation of relative specificity. (B) Resolution algorithm for evaluation of samples found repeatedly reactive during specificity testing. Resolution testing was performed at two independent reference centers. ELISA, enzyme-linked immunosorbent assay; IF, immunofluorescence.

Initially reactive (IR) samples with incomplete retesting, or without retesting, were considered RR. IR and RR gray-zone samples for the Abbott Architect Chagas assay were considered reactive. Furthermore, an aliquot of discrepant and concordant reactive samples was stored for further resolution testing at two reference centers according to their local diagnostic algorithms (representing the surrogate “gold standard”) (see below and Fig. 1).

### Analytical specificity.

The analytical specificity of the Elecsys Chagas assay was evaluated at two sites using potentially cross-reacting samples for other infectious diseases (see Table 4), e.g., leishmaniasis, malaria, Epstein-Barr virus, dengue virus, syphilis, toxoplasmosis, and human African trypanosomiasis.

### Analytical sensitivity at the cutoff.

Serial dilutions of two anti-T. cruzi antibody preparations from the National Institute for Biological Standards and Control (NIBSC) were measured in a single run (single determination per sample) by using the Elecsys Chagas assay and comparison assays. The WHO 1st International Standard for Chagas (TcI) antibody in human plasma (NIBSC panel 09/188) freeze-dried preparation contains anti-T. cruzi antibodies and consists of seropositive samples from autochthonous individuals living in Mexico, the region where T. cruzi genotype I is endemic. The WHO 1st International Standard for Chagas (T. cruzi genotype II [TcII]) antibody in human plasma (NIBSC panel 09/186) freeze-dried preparation contains anti-T. cruzi antibodies and is representative of seropositive samples from autochthonous individuals living in Brazil, the region where T. cruzi II is endemic.

Each standard was dissolved in deionized water to a final concentration of 0.5 IU/ml. Serial 1:2 pool dilutions were performed by using Chagas-negative serum and distributed to the laboratories for analysis (dilutions ranged from 1:2 to 1:8,192, corresponding to theoretical concentrations of 250 to 0.0610 mIU/ml of the respective antibody standards).

Comparison assays were the Abbott Prism Chagas assay, the Abbott Architect Chagas assay, the DiaSorin Liaison XL Murex Chagas assay, the Wiener Lab Chagatest, the Ortho T. cruzi ELISA for whole-cell lysates, the NovaTech NovaLisa Chagas assay, and the Biokit Bioelisa Chagas IgG assay.

### Confirmatory testing: resolution testing.

Discrepant and concordant reactive results from relative specificity testing underwent resolution testing at two independent reference centers using Conformité Européenne-labeled or in-house methods representing state-of-the-art Chagas antibody assays (Fig. 1). The final interpretation of the result for each sample was used as the basis for the assessment of relative specificity.

### Confirmatory testing: neutralization testing.

If sufficient sample volumes remained after resolution testing (Fig. 1), discrepant Elecsys Chagas results for reactive samples from the blood donor cohort were retested by using an in-house neutralization method similar to that reported previously (31). Briefly, the antigen extract (aqueous ultrasonic lysate supernatant) from native T. cruzi (CL Brener or DM28c) was added to the samples to a final concentration of 50 μg/ml T. cruzi antigen extract to generate a competitive situation between the recombinant antigens used in the Elecsys Chagas assay and the native T. cruzi antigen extract. After this pretreatment procedure was done to form stable antigen-antibody complexes, samples were subsequently rerun with the Elecsys Chagas assay. The obtained cutoff index (COI) values were compared with those derived by using the untreated sample, and the recovery of the neutralized sample was calculated. A recovery of ≤25% of the initial concentration was interpreted as reconfirmation of the presence of anti-T. cruzi antibodies.

### Data analyses.

Measurements were captured by using WinCAEv software; statistical analyses were performed by using R-package VCA (version 1.2.1) and SAS (version 9.3; SAS Institute). Interpretation of assay results was performed according to the package inserts of the respective assays.

Relative sensitivity and specificity, and analytical sensitivity, are expressed as point estimates and two-sided 95% confidence intervals (CIs). For the Elecsys Chagas assay, samples with a signal/cutoff (CO) ratio of ≥1.0 were considered reactive (i.e., positive for antibodies to T. cruzi), and those with a signal/CO ratio of <1.0 were considered nonreactive (i.e., negative for antibodies to T. cruzi). Results of the comparator assays were interpreted as follows: reactive if values were higher than or equal to the CO and nonreactive if values were lower than the CO for the whole-cell-lysate Ortho T. cruzi ELISA; reactive if signal/CO ratios were ≥1.0, gray zone if signal/CO ratios were ≥0.8 to <1.0, and nonreactive if signal/CO ratios were <0.8 for Abbott Architect Chagas; reactive if signal/CO ratios were ≥1.0 and nonreactive if signal/CO ratios were <1.0 for Abbott Prism Chagas; reactive if signal/CO ratios were ≥1.0 and nonreactive if signal/CO ratios were <1.0 for DiaSorin Liaison XL Murex Chagas; reactive if values were higher than or equal to the CO and nonreactive if values were lower than the CO for Wiener Lab Chagatest; reactive if values were higher than or equal to the CO for absorbance plus 10%, gray zone if values were ±10% of the CO signal, and negative if values were lower than the CO signal minus 10% for NovaTech NovaLisa Chagas; and positive if signal/CO ratios were ≥1.0, equivocal if signal/CO ratios were ≥0.9 to <1.0, and negative if signal/CO ratios were <0.9 for Biokit Bioelisa Chagas IgG.

## RESULTS

### Relative sensitivity.

A total of 674 precharacterized positive frozen samples were tested (Table 1). Precharacterization was performed via serology and PCR (*n* = 158) or via serology alone (*n* = 516), with and without clinical staging (*n* = 135 and *n* = 381, respectively) (Table 2).

**TABLE 2:** Characterization of the cohort used to determine relative sensitivity

Cohort of Chagas disease-positive samples (*n* = 674)	Tests used^*d*^
Regions of endemicity (*n* = 174)^*a*^	
Serological characterization (*n* = 174)	ELISA, HAI, antibodies induced by T. cruzi (anti-T. cruzi homogenate, anti-FRA, anti-p2β, anti-B13)
Serological characterization + clinical stage characterization (*n* = 135)^*b*^	ELISA, HAI, antibodies induced by T. cruzi (anti-T. cruzi homogenate, anti-FRA, anti-p2β, anti-B13), chest and abdominal X rays, electrocardiogram, echocardiogram
Regions of nonendemicity (*n* = 500)^*c*^	
Serological characterization (*n* = 342)	In-house CNM IFAT, in-house CNM ELISA, Wiener Lab Chagatest ELISA recombinante v.4.0
PCR + serological characterization (*n* = 158)	T. cruzi kDNA PCR, in-house CNM IFAT, in-house CNM ELISA, Wiener Lab Chagatest ELISA recombinante v.4.0

a Samples provided by the Consejo Nacional de Investigaciones Científicas y Técnicas (CONICET), Argentina.

b Chronic stage I (*n* = 66), stage II (*n* = 44), and stage III (*n* = 25) Chagas disease.

c Samples provided by the Instituto de Salud Carlos III, Spain.

d Anti-FRA, anti-flagellar repetitive antigen; IFAT, indirect fluorescent antibody technique; kDNA, kinetoplast DNA.

All Chagas disease-positive samples were correctly identified by the Elecsys Chagas assay (sensitivity of 100%; 95% CI, 99.45 to 100%). The Abbott Architect assay correctly identified all Chagas disease-positive samples (sensitivity, 100%; 95% CI, 97.90 to 100%; *n* = 174), while the Ortho T. cruzi assay detected positive samples with a sensitivity of 96% (95% CI, 93.89 to 97.54%; *n* = 500).

### Relative specificity.

A total of 14,681 samples from blood donors were tested (Fig. 1A); the relative specificities of the Elecsys Chagas assay (and comparison methods) after resolution testing (Fig. 1B) are summarized in Table 3. The Elecsys Chagas assay had overall relative specificities of 99.88% (for IR samples) (95% CI, 99.81 to 99.93%) and 99.90% (for RR samples) (95% CI, 99.83 to 99.94%). Relative specificities were 99.70% (for IR samples) (95% CI, 99.51 to 99.83%) and 99.74% (for RR samples) (95% CI, 99.56 to 99.86%) for the Latin American subgroup and 99.98% (for IR and RR samples) (95% CI, 99.93 to 100%) for the European subgroup. Overall, there were 26 qualitative discrepant results (*n* = 10 for Europe, and *n* = 16 for Latin America) versus the results of comparator assays, and 13 concordant reactive results (Latin America) were identified. The relative specificities (for RR samples) were 100% with the Ortho T. cruzi ELISA (95% CI, 99.93 to 100%) (*n* = 5,241), 99.96% with the DiaSorin Liaison assay (95% CI, 99.86 to 100%) (*n* = 5,244), 99.93% with the Abbott Prism assay (95% CI, 99.80 to 99.99%) (*n* = 4,391), and 99.78% with the Abbott Architect assay (95% CI, 99.61 to 99.89%) (*n* = 5,046).

**TABLE 3:** Relative specificity of the Elecsys Chagas assay in blood donor samples^*a*^

Parameter	Value for test				
	Elecsys Chagas	Ortho T. cruzi ELISA	DiaSorin Liaison XL Murex Chagas	Abbott Prism Chagas	Abbott Architect Chagas
Overall cohort					
Total no. of samples	14,681	5,241	5,244	4,391	5,046
No. of confirmed positive samples	8	0	0	0	8
No. of negative samples	14,673	5,241	5,244	4,391	5,038
No. of IR samples	25	0	2	6	15
No. of RR samples^*b*^	23	0	2	3	14
No. of RR samples confirmed positive/total no. of RR samples	8/23	0/0	0/2	0/3	8/14
Specificity (%) for IR samples (95% CI)	99.88 (99.81–99.93)	100 (99.93–100)	99.96 (99.86–100)	99.86 (99.70–99.95)	99.78 (99.61–99.89)
Specificity (%) for RR samples (95% CI)	99.90 (99.83–99.94)	100 (99.93–100)	99.96 (99.86–100)	99.93 (99.80–99.99)	99.78 (99.61–99.89)
European subgroup					
Total no. of samples	9,635	5,241	5,244	4,391	NA
No. of IR samples	2	0	2	6	NA
No. of RR samples	2	0	2	3	NA
No. of RR samples confirmed positive/total no. of RR samples	0/2	0/0	0/2	0/3	NA
Specificity (%) for IR samples (95% CI)	99.98 (99.93–100)	100 (99.93–100)	99.96 (99.86–100)	99.86 (99.70–99.95)	NA
Specificity (%) for RR samples (95% CI)	99.98 (99.93–100)	100 (99.93–100)	99.96 (99.86–100)	99.93 (99.80–99.99)	NA
Latin American subgroup					
Total no. of samples	5,046	NA	NA	NA	5,046
No. of IR samples	23	NA	NA	NA	15
No. of RR samples^*b*^	21	NA	NA	NA	14
No. of RR samples confirmed positive/total no. of RR samples	8/21	NA	NA	NA	8/14
Specificity (%) for IR samples (95% CI)	99.70 (99.51–99.83)	NA	NA	NA	99.78 (99.61–99.89)
Specificity (%) for RR samples (95% CI)	99.74 (99.56–99.86)	NA	NA	NA	99.78 (99.61–99.89)

a CI, confidence interval; IR, initially reactive; NA, not applicable; RR, repeatedly reactive.

b Thirteen reactive samples (in at least one assay) with incomplete retesting were considered RR for specificity calculations.

A total of 313 residual samples from pregnant women were tested. There were no discrepant or concordant reactive results. The Elecsys Chagas assay had an overall relative specificity of 100% (for IR and RR samples) (95% CI, 98.83 to 100%), which was generally consistent between the subgroups in the region of endemicity (Latin America) (100% for IR and RR samples [95% CI, 95.14 to 100%]) (*n* = 74 samples) and the region of nonendemicity (European) (100% for IR and RR samples [95% CI, 98.47 to 100%]) (*n* = 239 samples). Comparable results were obtained with the DiaSorin Liaison and Abbott Architect assays (data not shown).

A total of 517 residual samples from hospitalized patients were tested, and there were no discrepant or concordant reactive results. The Elecsys Chagas assay had an overall relative specificity of 100% (for IR and RR samples) (95% CI, 99.29 to 100%), which was generally consistent between the Latin American (100% for IR and RR samples [95% CI, 80.49 to 100%]) (*n* = 17 samples) and European (100% for IR and RR samples [95% CI, 99.26 to 100%]) (*n* = 500 samples) subgroups. Comparable results were obtained with the DiaSorin Liaison XL Murex Chagas and Abbott Architect assays (data not shown).

### Analytical specificity.

A total of 594 potentially cross-reactive samples were tested with the Elecsys Chagas assay (Table 4), and the overall analytical specificity was 99.83% (95% CI, 99.07 to 100%).

**TABLE 4:** Analytical specificity of the Elecsys Chagas assay with potentially cross-reactive samples^*a*^

Potentially cross-reacting condition or disease state^*b*^	Total no. of samples	No. (%) of nonreactive samples	No. (%) of reactive samples
Epstein-Barr virus	26	26 (100)	0
Dengue virus	87	87 (100)	0
Leishmaniasis	241	241 (100)	0
Malaria	204	203 (99.5)	1 (0.5)
Syphilis	19	19 (100)	0
Toxoplasmosis	15	15 (100)	0
Human African trypanosomiasis	2	2 (100)	0
Total	594	593	1

a Samples were tested at Roche Diagnostics Centralized and Point of Care Solutions (Penzberg, Germany), unless stated otherwise.

b A total of 164 Leishmania-positive and 100 malaria-positive serum/plasma samples were tested in Madrid, Spain; samples (serotheque) were previously stored frozen. Samples used for the analytical specificity study were derived from commercial vendors (Acess Biologicals, USA; Slieagen LLC, USA, Cerba Specimens Services, France; Trina Bioreactives AG, Switzerland; BioClinical Partner Inc., USA; and DiaServe GmbH, Germany), routine laboratories, and institutions (Instituto de Salud Carlos III, Madrid, Spain). Characterization of the samples was done by either serological analysis, parasitological certificate, or clinical definition.

A subgroup of precharacterized leishmaniasis-positive samples (*n* = 164) and malaria-positive samples (*n* = 100) from Spain were tested by both the Elecsys Chagas assay and the Ortho T. cruzi ELISA. In the leishmaniasis-positive cohort, all samples were nonreactive for T. cruzi antibodies with the Elecsys Chagas assay (analytical specificity of 100%; 95% CI, 97.78 to 100%), while 65 samples tested positive with the Ortho T. cruzi ELISA (analytical specificity of 60.37%; 95% CI, 52.44 to 67.91%). In the malaria-positive cohort, one sample was reactive with the Elecsys Chagas assay (low-level COI of 1.11; analytical specificity of 99.00%; 95% CI, 94.55 to 99.97%) and four samples tested positive with the Ortho T. cruzi ELISA (analytical specificity of 96.00%; 95% CI, 90.07 to 98.90%). Plasmodium-T. cruzi coinfection was ruled out. These samples were obtained from Spanish citizens and immigrant residents who had traveled to South Asia and/or Africa.

Six additional samples (*n* = 5 for dengue, and *n* = 1 for leishmaniasis) were excluded from the analysis because a coinfection could not be ruled out. These samples originated from a region where Chagas disease is endemic (Argentina) and were found to be reactive in the Elecsys Chagas assay (with COI values ranging from 13.3 to 206) as well as in at least one additional Chagas antibody assay. Four out of five dengue samples thereof were also found to be highly reactive in at least two comparator Chagas assays. Further resolution testing was not possible due to a lack of sample volume.

### Analytical sensitivity at the cutoff.

The analytical sensitivities at the cutoff of the Elecsys Chagas assay and comparator assays (*n* = 3 automated assays, and *n* = 4 nonautomated ELISAs, reflecting local routine methods) were assessed by using two WHO standard NIBSC reference panels. The Elecsys Chagas assay detected T. cruzi antibodies at a 1:512 dilution for both reference panels (Table 5), corresponding to a cutoff sensitivity of approximately 1 mIU/ml. Comparison assays were found reactive at dilutions of 1:32 for panel 09/188 (T. cruzi I) and 1:16 for panel 09/186 (T. cruzi II), corresponding to cutoff sensitivities of 15.6 mIU/ml and 31.3 mIU/ml, respectively, or less sensitivity.

**TABLE 5:** Detection limits of the Elecsys Chagas and comparison assays using World Health Organization standard National Institute for Biological Standards and Control reference panels 09/188 (T. cruzi I) and 09/186 (T. cruzi II)^*a*^

Dilution	Concn (mIU/ml)	Reactivity (signal/CO)							
		Elecsys Chagas	Abbott Prism Chagas	Abbott Architect Chagas	DiaSorin Liaison XL Murex Chagas	Wiener Lab Chagatest	Ortho T. cruzi ELISA	NovaTech NovaLisa Chagas	Biokit Bioelisa Chagas
Panel 09/188									
1:8,192	0.06	0.14	0.06	0.26	0.03	0.20	0.31	0.52	
1:4,096	0.12	0.21	0.08	0.13	0.03	0.14	0.23	0.61	
1:2,048	0.24	0.32	0.06	0.21	0.03	0.19	0.25	0.56	0.04
1:1,024	0.49	0.57	0.07	0.19	0.03	0.20	0.38	0.61	0.04
1:512	0.98	**1.02**	0.07	0.20	0.03	0.20	0.38	0.53	0.07
1:256	1.95	**2.00**	0.11	0.31	0.04	0.26	0.36	0.53	0.02
1:128	3.91	**3.87**	0.14	0.47	0.05	0.34	0.38	0.54	0.07
1:64	7.81	**7.63**	0.17	*0.92*	0.08	0.49	0.55	0.55	0.10
1:32	15.6	**15.1**	0.95	**2.02**	0.17	0.69	0.85	0.63	0.13
1:16	31.3	**30.4**	**1.19**	**3.17**	0.36	**1.00**	**1.11**	0.81	0.15
1:8	62.5	**58.8**	**2.10**	**5.14**	0.73	**1.68**	**1.78**	*0.99*	0.46
1:4	125	**118**	**2.89**	**6.57**	**1.50**	**2.63**	**2.58**	**1.60**	0.84
1:2	250	**209**	**5.64**	**8.32**	**2.80**	**3.44**	**3.81**	**2.34**	**1.97**
Undiluted	500	**246**	**6.25**	**9.78**	**4.90**	**4.37**	**4.78**	**3.37**	**1.41**
Panel 09/186									
1:8,192	0.06	0.15	0.06	0.14	0.03	0.22	0.27	0.55	
1:4,096	0.12	0.21	0.05	0.14	0.03	0.21	0.32	0.64	
1:2,048	0.24	0.34	0.06	0.13	0.03	0.21	0.59	0.58	0.06
1:1,024	0.49	0.61	0.07	0.14	0.03	0.23	0.43	0.54	0.06
1:512	0.98	**1.10**	0.06	0.19	0.03	0.21	0.33	0.54	0.05
1:256	1.95	**2.08**	0.06	0.20	0.03	0.27	0.31	0.54	0.06
1:128	3.91	**4.00**	0.09	0.27	0.04	0.29	0.30	0.52	0.08
1:64	7.81	**7.89**	0.09	0.37	0.04	0.38	0.41	0.56	0.12
1:32	15.6	**15.6**	0.10	0.74	0.07	0.73	0.50	0.60	0.16
1:16	31.3	**30.7**	0.27	**1.49**	0.11	0.95	0.87	0.70	0.27
1:8	62.5	**60.2**	0.95	**2.54**	0.27	**1.43**	**1.41**	*0.92*	0.51
1:4	125	**112**	**2.84**	**4.56**	0.54	**2.54**	**1.92**	*1.09*	**1.01**
1:2	250	**192**	**2.21**	**6.52**	**1.10**	**3.41**	**2.96**	**1.76**	**1.86**
Undiluted	500	**229**	**5.04**	**8.49**	**1.90**	**4.43**	**4.12**	**2.95**	**1.50**

a Data in boldface type represent reactive results; lightface type, nonreactive results. Data in italic type represent results within the “gray zone” for the NovaTech NovaLisa or the Abbott Architect assay.

### Typical distribution of values.

**FIG 2:**
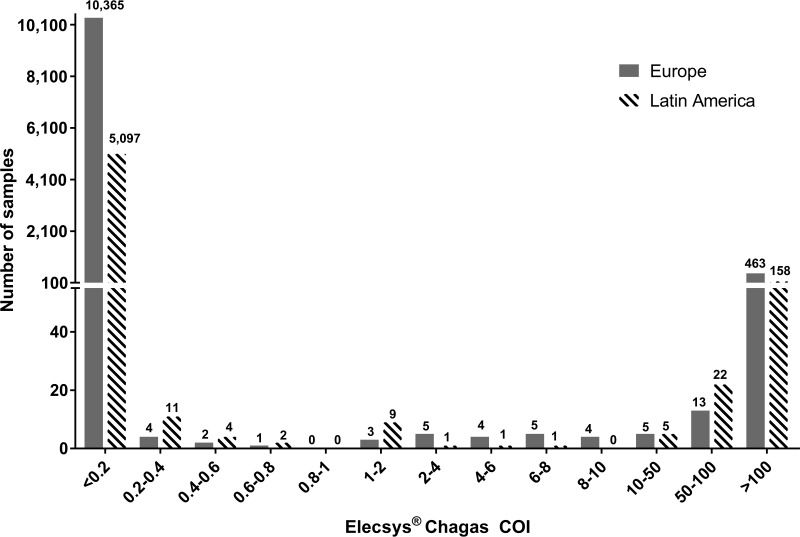
Distribution of COI values for reactive and nonreactive samples from blood donors, pregnant women, hospitalized patients, and Chagas-positive patients, measured with the Elecsys Chagas assay (*n* = 16,185) (suitable COI increments were chosen).

The distribution of COI values for 16,185 samples (reactive and nonreactive samples from blood donors, pregnant women, hospitalized patients, and confirmed Chagas disease-positive cases) is displayed in Fig. 2. The Elecsys Chagas assay revealed good discrimination between reactive and nonreactive samples. Only a small number of the samples were found to have low positive COI values in the Elecsys Chagas assay (*n* = 16,185; 12 samples with COI values ranging from 1 to 2, representing 0.07%) (Fig. 2).

The lowest COI values observed for the precharacterized Chagas disease-positive cohort (*n* = 674) were 1.79 and 1.80, thus representing just 0.3% of the total positive-sample cohort. All other Chagas disease patient samples showed COI values ranging from 2.3 to >300.

### Neutralization.

A total of 10 Elecsys assay-discrepant RR samples from the blood donor cohort underwent in-house neutralization testing to further assess the presence of antibodies to T. cruzi that might be undetectable by comparator methods with lower sensitivities. Five samples from regions of endemicity were successfully neutralized (≤25% recovery of the COI), and one sample from a region of endemicity showed a borderline tendency toward neutralization with the native antigen pretreatment procedure (26% recovery). Four samples could not be neutralized and revealed 28 to 98% COI recoveries (Table 6).

**TABLE 6:** Neutralization results for Elecsys Chagas assay-discrepant, repeatedly reactive samples from blood donors

Study site	Comparator COI	Elecsys COI^*a*^	Elecsys COI after neutralization	Recovery^*b*^ (%)
Italy	0.016	2.94	1.87	64
Colombia	0.023	1.16	0.298	26
Colombia	0.037	1.09	0.300	28
Colombia	0.554	1.47	0.515	35
Argentina	0.230	12.6	0.348	**3**
Argentina	0.99/0.97/1.14^*c*^	40.8	2.04	**5**
Argentina	0.040	2.92	0.227	**8**
Argentina	0.080	1.60	0.132	**8**
Argentina	0.510	1.47	0.152	**10**
Argentina	0.030	1.03	1.01	98

a COI as determined by Roche Diagnostics Centralized and Point of Care Solutions (Penzberg, Germany) prior to the neutralization procedure.

b A recovery of ≤25% was assessed as successful neutralization (i.e., positive for anti-T. cruzi antibodies). Values in boldface type represent neutralizable/borderline and neutralizable samples.

c The COI was repeatedly in the gray zone.

## DISCUSSION

In the present study, the Elecsys Chagas assay demonstrated excellent analytical performance in a multicenter study in Europe and Latin America compared with established assays. Although the Elecsys Chagas assay uses a new combination of just three different recombinant T. cruzi antigens, the performance observed in the present study supports its use as a diagnostic and screening test. Since screening for blood products harboring T. cruzi is a critical component for blood safety, we also investigated the performance of the test on samples from blood donors from various regions of endemicity and nonendemicity. Importantly, a substantial number of serum samples from patients known to have Chagas disease from different regions were included in the study. Furthermore, the excellent analytical specificity was confirmed by using a large panel of potentially cross-reactive samples or samples from individuals with other infectious diseases. Finally, the performance of the Elecsys Chagas assay was also verified with samples from pregnant women and hospitalized patients, and the results were comparable, irrespective of whether the samples were sourced from regions of endemicity or from regions of nonendemicity.

A number of studies showed various T. cruzi antigens (either native or recombinant or as peptide or multiepitope antigen) to potentially be suitable for use as serodiagnostic tools (32–37). However, the numbers of samples used for evaluation varied, and the statistical power of the results may be limited. Studies including a significant number of blood donor screening samples, potentially cross-reacting samples, and proven reactive samples are found less frequently (20, 38–41). WHO-driven comparison activities were conducted over a decade ago (42), with specificity and sensitivity varying considerably among the 18 screening assays evaluated. A definitive resolution of the discrepant findings was difficult since “consensus positives” and “consensus negatives” always inherit a selection bias, while the true serological status cannot be revealed (42). It may now be timely to conduct comparative evaluations of the new assays that have become available since that WHO study was reported, to ascertain their relative efficacies.

The WHO seeks to promote the identification of novel diagnostic tests for Chagas disease (1). Although there are a variety of methods available to confirm the presence of T. cruzi infection (ELISA-based methods, immunofluorescence-based methods, immunoblotting, PCR, and microscopy), there is currently no gold standard, and testing of samples with multiple assays is often necessary, creating a combined gold standard as a surrogate (13, 17, 28). This leaves manufacturers of new, highly sensitive tests with a dilemma.

In the present study, the surrogate gold standard against which the Elecsys assay was compared included at least three serological assays or PCR (for the evaluation of relative sensitivity) and resolution testing at two independent reference centers using several commercial CE-labeled tests and well-evaluated in-house assays representing state-of-the-art methods (for the evaluation of relative specificity).

Our study results support the notion that existing methods worldwide may not be adequate to confirm the results for samples with low-level antibody concentrations, which may be best confirmed according to the epidemiological and clinical background. Equally, the results for samples with higher concentrations in this study could potentially be confirmed with any test. Moreover, our findings add to the evidence suggesting that single assays with improved sensitivity and specificity may be sufficient for screening and diagnosis purposes, respectively (28).

Since the Elecsys Chagas assay was developed without a gray zone, and a clear separation of reactive versus nonreactive samples was validated in this multicenter evaluation, the potential number of unclear and inconclusive results or “low-titer” samples following the initial analysis is expected to be reduced significantly compared with previously described assays (28) or resolution algorithms (27). A highly sensitive automated method to screen for Chagas disease, such as the Elecsys Chagas assay, could potentially increase the throughput of samples and will likely lead to improvements in diagnosis algorithms and, thus, in cost-effectiveness (28). Ultimately, the availability of improved assays for the detection of T. cruzi would be expected to better safeguard patients who require blood and organ donation and help to minimize misdiagnoses, which are major factors in delaying the appropriate health care response (21).

Strengths of this study are the inclusion of a significant proportion of samples from Latin America to evaluate the Elecsys Chagas assay under blood screening and diagnostic routine laboratory conditions in countries where the disease is endemic. This is important because geographical differences in the sensitivities of recombinant antigen-based rapid tests for T. cruzi infection have been demonstrated, possibly due to T. cruzi strain differences (43). Commercially available performance panels covering samples from an additional nine countries were all found to be reactive with the Elecsys assay (Roche internal data [not shown]), underlining the sensitivity for Chagas samples from South and Central America. The present study also included an analysis of reactive samples stored frozen for a period of years, demonstrating the general stability of the analyte (IgG *per se*). Moreover, in samples that showed a loss of reactivity with competitor assays during long-term storage, the Elecsys COI values ranged from 1.8 to 70.9, supporting the high sensitivity of this assay. Finally, the Elecsys Chagas assay was compared with several existing assays to ensure relevance to local protocols and thus to current benchmarks for performance. Evaluation with a commercially available seroconversion panel revealed a seroconversion sensitivity identical to those of competitors, thus reconfirming the sensitivity for samples derived from the early phase of infection (Table 7).

**TABLE 7:** Evaluation of seroconversion sensitivity with a commercially available seroconversion panel, SeraCare Chagas (T. cruzi) AccuVert seroconversion panel 0615-0038

Panel member	Bleed date (day.mo.yr)	No. of days since 1st bleed	Roche Diagnostics Elecsys Chagas signal/CO ratio	Interpretation of result
1	31.07.2012	0	0.071	Nonreactive
2	10.09.2012	41	117	Reactive
3	17.09.2012	48	118	Reactive
4	24.09.2012	55	127	Reactive
5	01.10.2012	62	143	Reactive
6	08.10.2012	69	151	Reactive
7	15.10.2012	76	146	Reactive
8	29.10.2012	90	178	Reactive
9	12.11.2012	104	169	Reactive
10	26.11.2012	118	210	Reactive

Compared with the comparator tests, discrepant results were observed for 26 of 14,681 blood donor samples (0.17%) derived from regions of nonendemicity and endemicity, a significantly lower percentage than those observed with new-generation competitors (28). Since there is no established gold standard for the detection of anti-T. cruzi antibodies, we used a neutralization test to further investigate such discrepant reactive results obtained with the Elecsys test. The application of a neutralization test for the verification of discrepant reactive results in a highly sensitive assay to detect anti-T. gondii antibodies was described previously (31) and was successfully applied here for T. cruzi. The relative specificity (99.74%) observed in the present study for the subgroup of blood donors from Latin America may therefore be even higher due to the reconfirmed presence of specific antibodies in Elecsys-discrepant reactive samples. The in-house neutralization method to resolve discrepant reactive findings with state-of-the-art assays was used here for the first time within a multicenter study and was deemed to be an additional specific and valuable method. The use of the heterologous native T. cruzi antigen extract for supplemental neutralization testing avoids an inbreeding confirmatory situation for the recombinant antigens used by the Elecsys assay. However, we were unable to perform neutralization testing on all samples with qualitative discrepant results due to a lack of sufficient sample volumes in some cases. Due to the use of residual blood donor samples, there was also no possibility of serological donor follow-up to clarify questionable results.

The high analytical sensitivity of the Elecsys assay is reflected in comparisons of cutoff sensitivities based on the use of material accessible to the public, such as WHO reference material from the NIBSC. Such reference material may help to better benchmark the dilutional sensitivities and the individual cutoff settings of the respective assays. This approach is widely used to assess the performance of screening assays and is also an inherent part of the Common Technical Specifications (CTS) of European Commission directive 98/79 for screening assays (45). The recombinant assay format described here is highly sensitive, which contradicts the notion that only techniques that use whole parasites are sufficiently sensitive (44). Sensitivity assessment regarding different distinct typing units of T. cruzi was covered during the development of the assay by an analysis of 1,370 suspected Chagas disease-positive samples (all investigated samples were reactive in ≥3 assays) derived from 13 countries (Argentina, Bolivia, Chile, Spain, Ecuador, El Salvador, Honduras, Mexico, Nicaragua, Paraguay, Uruguay, the United States, and Venezuela), revealing 100% reactive results with the Elecsys Chagas assay (Roche internal data [not shown]) (the lowest observed signal/CO value for all samples was >2). Thus, the assay is suitable for blood donor management as well as for diagnostic use.

The development of highly sensitive and specific new assays for the detection of anti-T. cruzi antibodies thus helps to reduce expenses for additional second-line testing for the diagnosis of the disease and safeguards the sensitivity needed for blood screening purposes.

### Conclusions.

The automated Elecsys Chagas assay demonstrated a robust and favorable performance under routine conditions at multiple sites in Europe and Latin America. In contrast to other available assays for Chagas disease, the Elecsys assay uses a reduced number of recombinant T. cruzi antigens, resulting in a significantly smaller number of cross-reactions, with improved analytical specificity, while still being highly sensitive, with high discriminatory power.
